# Admission fasting plasma glucose is associated with in-hospital outcomes in patients with acute coronary syndrome and diabetes: findings from the improving Care for Cardiovascular Disease in China - Acute Coronary Syndrome (CCC-ACS) project

**DOI:** 10.1186/s12872-020-01662-3

**Published:** 2020-08-20

**Authors:** Nan Ye, Lijiao Yang, Guoqin Wang, Weijing Bian, Fengbo Xu, Changsheng Ma, Dong Zhao, Jing Liu, Yongchen Hao, Jun Liu, Na Yang, Hong Cheng, on behalf of the CCC-ACS

**Affiliations:** 1 Renal Division, Beijing Anzhen Hospital, Capital Medical University, Beijing, China; 2 Department of Cardiology, Beijing Anzhen Hospital, Capital Medical University, Beijing, China; 3 Department of Epidemiology, Beijing Anzhen Hospital, Capital Medical University, Beijing Institute of Heart, Lung and Blood Vessel Diseases, No. 2 Anzhen Street, Chao yang District, Beijing, 100029 PR China

**Keywords:** Acute coronary syndrome, Diabetes, Fasting plasma glucose, Glycosylated hemoglobin

## Abstract

**Background:**

The discrepancy between glycosylated hemoglobin (HbA_1c_) and fasting plasma glucose (FPG) in clinical practice may be related to factors such as acute stress, renal dysfunction, and anemia, and its relationship with in-hospital outcomes is uncertain. The aim of this study was to investigate the association between the type of discrepancy between HbA_1c_ and FPG and in-hospital outcomes in patients with acute coronary syndrome (ACS) and diabetes.

**Methods:**

The Improving Care for Cardiovascular Disease in China - Acute Coronary Syndrome (CCC-ACS) project is a national, hospital-based quality improvement project with an ongoing database. Patients with ACS, diabetes and complete HbA_1c_ and FPG values at admission were included. The consistent group included patients with HbA_1c_ < 6.5% and FPG < 7.0 mmol/L or HbA_1c_ ≥ 6.5% and FPG ≥ 7.0 mmol/L. The discrepancy group included patients with HbA_1c_ ≥ 6.5% and FPG < 7.0 mmol/L (increased HbA_1c_ group) or HbA_1c_ < 6.5% and FPG ≥ 7.0 mmol/L (increased FBG group).

**Results:**

A total of 7762 patients were included in this study. The numbers of patients in the consistent and discrepancy groups were 5490 and 2272 respectively. In the discrepancy group, increased HbA_1c_ accounted for 77.5% of discrepancies, and increased FPG accounted for 22.5% of discrepancies. After adjusting for confounders, patients in the increased FPG group had a 1.6-fold increased risk of heart failure (OR, 1.62; 95% CI, 1.08–2.44), a 1.6-fold increased risk of composite cardiovascular death and heart failure (OR, 1.63; 95% CI, 1.09–2.43), and a 1.6-fold increased risk of composite major adverse cardiovascular and cerebrovascular events (MACCEs) and heart failure (OR, 1.56; 95% CI, 1.08–2.24) compared to patients in the increased HbA_1c_ group.

**Conclusions:**

Patients with an increased FPG but normal HbA_1c_ had a higher risk of in-hospital adverse outcomes than those with increased HbA_1c_ but normal FPG. This result may indicate that when HbA_1c_ and FPG are inconsistent in patients with ACS and diabetes, the increased FPG that may be caused by stress hyperglycemia may have a more substantial adverse effect than increased HbA_1c_, which may be caused by chronic hyperglycemia. These high-risk patients should be given more attention and closer monitoring in clinical practice.

**Trial registry:**

Clinicaltrial.gov, NCT02306616. Registered 29 November 2014.

## Background

Cardiovascular disease is the leading cause of both death and premature death in China and is the cause of 40% of deaths in the Chinese population [1]. Acute coronary syndrome (ACS) is an acute manifestation of cardiovascular disease with a high risk of mortality that can lead to critical conditions such as cardiogenic shock and cardiac arrest. Patients with ACS and diabetes usually have worse clinical outcomes than those with normal blood glucose [2–8], regardless of in-hospital or long-term outcomes. Indicators commonly used for evaluating blood glucose include intravenous blood glucose, glycosylated hemoglobin (HbA_1c_), and glycosylated serum albumin. Glucose was first used in the diagnosis of diabetes, including fasting plasma glucose (FPG), oral glucose tolerance tests and random blood glucose. In 2013, the American Diabetes Association approved the use of HbA_1c_ to diagnose diabetes [9]. In addition to their diagnostic value, FPG and HbA_1c_ are also associated with clinical outcomes. Several studies have shown that abnormal blood glucose is an important factor associated with clinical outcomes in patients with ACS and diabetes [10–17].

However, the discrepancy between HbA1c and FPG can be observed in clinical practice, which has not been fully investigated until now. This condition may be related to factors such as acute stress, renal dysfunction, and anemia, which may affect FPG and HbA_1c_. The discrepancy can be an increased FPG with a normal HbA_1c_ or an increased HbA_1c_ with a normal FPG. We decided to explore which discrepancy indicates worse in-hospital outcomes. There are few studies focusing on this issue.

The Improving Care for Cardiovascular Disease in China - Acute Coronary Syndrome (CCC-ACS) project is a national, hospital-based quality improvement project with an ongoing database, aiming to increase adherence to ACS guidelines in China and to improve patient outcomes. We conducted this study based on the CCC-ACS project to investigate the types of discrepancies between HbA_1c_ and FPG and their relationships to in-hospital outcomes.

## Methods

### Research design

Details of the design and methodology of the CCC-ACS project have been published [18], and the study was registered at ClinicalTrials.gov (NCT02306616). In brief, the CCC-ACS is a national, hospital-based quality improvement project with an ongoing database, aiming to increase adherence to ACS guidelines in China and to improve patient outcomes. It was launched in 2014 as a collaborative initiative of the American Heart Association and the Chinese Society of Cardiology. A total of 240 hospitals were recruited, representing the diversity of ACS care in hospitals in China, including 150 tertiary hospitals in phase I and phase II and 82 secondary hospitals and 8 tertiary hospitals in phase III (from July 2017) and phase IV (from November 2018). Clinical data were collected via a web-based data collection platform (Oracle Clinical Remote Data Capture, Oracle Corporation). Trained data abstractors entered the data elements abstracted from medical charts. Eligible patients were consecutively reported to the CCC-ACS database for each month before the middle of the following month. Approximately 5% of reported cases were randomly selected and compared with the original medical records. An audit by a third party was performed to ensure that cases were reported consecutively rather than selectively.

### Research population

A total of 104,516 inpatients with ACS, identified based on their principal diagnosis at discharge, were enrolled from 240 hospitals across China from November 2014 to December 2019. Patients with diabetes and complete HbA_1c_ and FPG values at admission were included in this study. Only patients from July 2017 to December 2019 were included in this study because the FPG value at admission was not included in the database before July 2017. Patients were divided into a consistent group and a discrepancy group based on the HbA_1c_ and FPG values at admission. The consistent group included patients with HbA_1c_ < 6.5% and FPG < 7.0 mmol/L or patients with HbA_1c_ ≥ 6.5% and FPG ≥ 7.0 mmol/L. The discrepancy group included patients with HbA_1c_ ≥ 6.5% and FPG < 7.0 mmol/L or patients with HbA1c < 6.5% and FPG ≥ 7.0 mmol/L. The discrepancy group was further divided into an increased HbA_1c_ but normal FPG group (HbA_1c_ ≥ 6.5% and FPG < 7.0 mmol/L) and an increased FPG but normal HbA_1c_ group (HbA_1c_ < 6.5% and FPG ≥ 7.0 mmol/L). Institutional review board approval was granted for the use of an aggregate data set for research and quality improvement by the Ethics Committee of Beijing Anzhen Hospital, Capital Medical University. No informed consent was required.

### Definition of variables

Diabetes was defined as having a history of diabetes, receiving glucose-lowering agents before hospitalization, having diabetes listed in the medical records as a secondary discharge diagnosis, or having HbA_1c_ ≥ 6.5% at admission. Hypertension was defined as having a history of hypertension, receiving antihypertensive medication, or having systolic blood pressure ≥ 140 mmHg or diastolic blood pressure ≥ 90 mmHg at admission. The ACS classification was based on the primary diagnosis at discharge in the medical record. Non-ST-segment elevation ACS was defined as non-ST-segment elevation myocardial infarction (STEMI) or unstable angina. All the laboratory testing values were the values tested the first time after admission. The estimated glomerular filtration rate (eGFR) was calculated according to the equation developed by the Chronic Kidney Disease Epidemiology Collaboration [19]. Medication was prescribed after admission.

### In-hospital outcomes

The outcomes of this study included major adverse cardiovascular and cerebrovascular events (MACCEs), heart failure, composite of cardiovascular death and heart failure, composite of MACCEs and heart failure, and death from any cause. MACCEs were defined as cardiovascular death, cardiac arrest, cardiogenic shock, recurrent myocardial infarction, stent thrombosis, and stroke.

### Statistical analysis

Continuous variables were presented as the mean and standard deviation or median and interquartile range when the distribution and variance met the appropriate conditions. Categorical variables were presented as percentages. The comparisons between groups of continuous variables were performed by an unpaired t-test or a Mann-Whitney U test (Kruskal-Wallis), and the chi-square test was used to compare the categorical variables. A multivariate logistic regression model was used to determine the association between the type of discrepancy and in-hospital outcomes by controlling for potential confounders. Candidate adjustment factors included age, gender, systolic blood pressure, heart rate, current smoker, hypertension, hemoglobin at admission, eGFR at admission, Killip class, type of acute coronary syndrome, glucose-lowering drug use, and β-blocker use during hospitalization. The heterogeneity of effects on the in-hospital outcomes across subgroups was estimated using random effects meta-analysis. For data with missing values lower than 15% (Additional file 1: Table S1), the sequential regression multiple imputation method implemented by IVEware software version 0.2 (Survey Research Center, University of Michigan, Ann Arbor, MI, USA) was used to impute the missing values. All *P* values were 2-tailed, and *P* <  0.05 was considered statistically significant. Statistical analyses were performed using SPSS 23.0 (SPSS Inc., Chicago, IL) and Stata/IC 15.1.

## Results

### Characteristics of patients in the discrepancy group

A total of 7762 patients were included in this study (Additional file 1: Figure S1). The mean age was 64.4 (±11.6) years, and males accounted for 68.8% of the study population. The mean hemoglobin level was 135.1 (± 21.3) g/L, and the mean eGFR was 81.7 (± 25.4) ml min^− 1^ (1.73 m)^− 2^. A total of 53.3% of patients were treated with at least one class of oral glucose-lowering drugs or insulin. The numbers of patients in the consistent group and the discrepancy group were 5490 (70.7%) and 2272 (29.3%), respectively. Patients in the discrepancy group were more likely to have lower eGFR (Additional file 1: Figure S2). In the discrepancy group, increased HbA_1c_ but normal FPG accounted for 77.5% of the discrepancies (1761/2272), and increased FPG but normal HbA_1c_ accounted for 22.5% of the discrepancies (511/2272). The baseline characteristics of patients in the increased HbA_1c_ but normal FPG group and increased FPG but normal HbA_1c_ group are shown in Table 1. Patients in the increased FPG but normal HbA_1c_ group were more likely to have lower eGFR, higher heart rate, poorer heart function, STEMI and hypertension and to be treated with glucose-lowering agents.

**Table 1:** Characteristics of patients with discrepancies between HbA_1c_ and FPG

	HbA1c ≥ 6.5% and FPG < 7.0 mmol/L (*n* = 1761)	HbA1c < 6.5% and FPG ≥ 7.0 mmol/L (*n* = 511)	*P* value
Age (years, mean [SD])	65.4(11.2)	65.4(10.9)	0.947
Male (n [%])	1184(67.2)	348(68.1)	0.713
Systolic blood pressure (mmHg, mean [SD])	135.0(22.8)	135.0(25.2)	0.895
Diastolic blood pressure (mmHg, median [IQR])	78.0(70.0, 87.0)	78.0(70.0, 89.0)	0.442
Heart rate (bpm. Median [IQR])	78.0(68.0, 87.0)	80.0(70.0, 90.0)	< 0.001
Current smoker (n [%])	508(28.8)	153(29.9)	0.047
Family history of CHD (n [%])	71(4.0)	21(4.1)	0.937
Hypertension (n [%])	1300(73.8)	403(78.9)	0.021
Previous acute myocardial infarction (n [%])	214(12.2)	58(11.4)	0.623
Previous coronary artery bypass grafting (n [%])	16(0.9)	4(0.8)	0.789
Atrial fibrillation history (n [%])	53(3.0)	14(2.7)	0.751
Heart failure history (n [%])	58(3.3)	19(3.7)	0.640
Cerebrovascular disease history (n [%])	185(10.5)	57(11.2)	0.675
Peripheral artery disease history (n [%])	31(1.8)	10(2.0)	0.769
Killip class (n [%])			0.289
I or II	1505(85.5)	427(83.6)	
III or IV	256(14.5)	84(16.4)	
Types of ACS (n [%])			< 0.001
STEMI	716(40.7)	276(54.0)	
NSTE-ACS	1045(59.3)	235(46.0)	
HbA_1c_ (%, mean [SD])	8.2(14.7)	5.8(0.8)	< 0.001
FPG (mmol/L, mean [SD])/(mg/dl, mean [SD])	5.6(1.2)/100.8(21.6)	9.5(2.6)/171.0(46.8)	< 0.001
eGFR (ml min^−1^ [1.73 m]^−2^, mean [SD])	79.1(25.1)	76.4(27.3)	0.046
Hemoglobin (g/l, mean [SD])	132.1(20.4)	132.4(23.6)	0.192
Total cholesterol (mmol/L, median [IQR])/(mg/dl, median [IQR])	4.3(3.5, 5.1)/166.2(135.3, 197.2)	4.3(3.5, 5.1)/166.2(135.3, 197.2)	0.721
HDL-cholesterol (mmol/L, median [IQR])/(mg/dl, median [IQR])	1.0(0.8, 1.2)/38.7(30.9, 46.4)	1.0(0.8, 1.2)/38.7(30.9, 46.4)	0.931
LDL-cholesterol (mmol/L, median [IQR])/(mg/dl, median [IQR])	2.6(2.0, 3.2)/100.5(77.3, 123.7)	2.5(2.0, 3.2)/96.7(77.3, 123.7)	0.326
Triglyceride (mmol/L, median [IQR])/(mg/dl, median [IQR])	1.6(1.1, 2.4)/141.8(97.5, 212.6)	1.5(1.0, 2.3)/132.9(88.6, 203.8)	0.027
Oral glucose-lowering agents or insulin use before admission (n [%])	809(45.9)	288(56.4)	< 0.001
Therapy during hospitalization (n [%])			
Percutaneous coronary intervention	1170(66.4)	337(65.9)	0.836
Aspirin	1644(93.4)	482(94.3)	0.432
P2Y_12_ inhibitors	1603(91.0)	473(92.6)	0.276
Statins	1653(93.9)	477(93.3)	0.669
β-blockers	1146(65.1)	291(56.9)	0.001
ACE inhibitor/angiotensin receptor blocker	917(52.1)	248(48.5)	0.159
Patients with referral (n [%])	587(33.3)	172(33.7)	0.891

*ACE* Angiotensin-converting enzyme, *ACS* Acute coronary syndrome, *CHD* Coronary heart disease, *eGFR* Estimated glomerular filtration rate, *FPG* Fasting plasma glucose, *HbA*_*1c*_ Glycosylated hemoglobin, *HDL* High-density lipoprotein, *IQR* Interquartile range, *LDL* Low-density lipoprotein, *NSTE-ACS* Non-ST-segment elevation acute coronary syndrome, *SD* Standard deviation, *STEMI* ST-segment elevation myocardial infarction

**Fig. 1:**
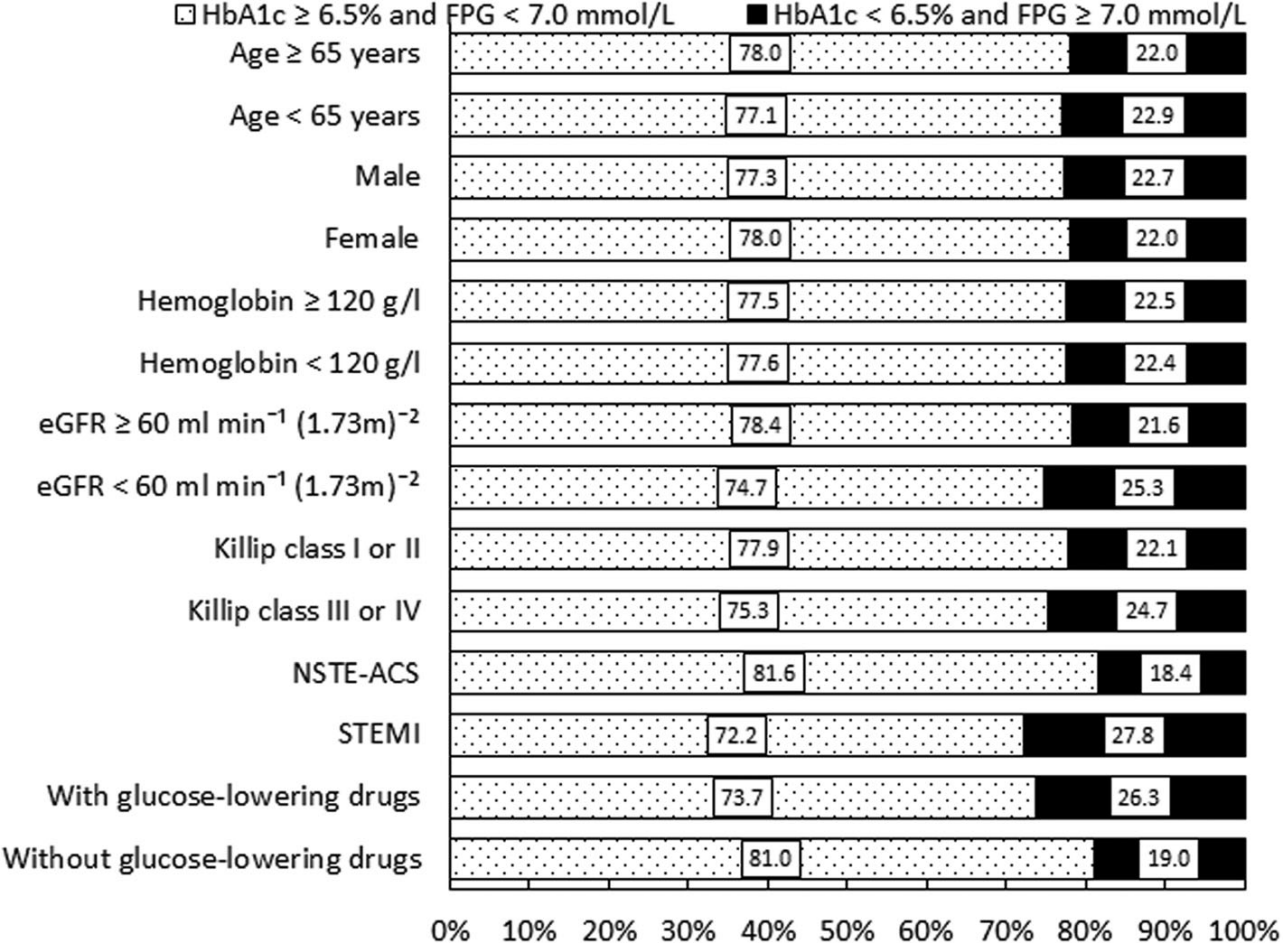
Prevalence of discrepancy in different population. eGFR, estimated glomerular filtration rate; FPG, fasting plasma glucose; HbA_1c_, glycosylated hemoglobin; NSTE-ACS, non-ST-segment elevation acute coronary syndrome; STEMI, ST-segment elevation myocardial infarction

The prevalence of the increased FPG but normal HbA_1c_ discrepancy was higher in patients over 65 years of age, hemoglobin less than 120 g/L, eGFR less than 60 ml min^− 1^ (1.73 m)^− 2^, Killip class III or IV, and STEMI and who were treated with oral glucose-lowering drugs or insulin (Fig. 1, Additional file 1: Table S2).

### Types of discrepancy and in-hospital outcomes

**Fig. 2:**
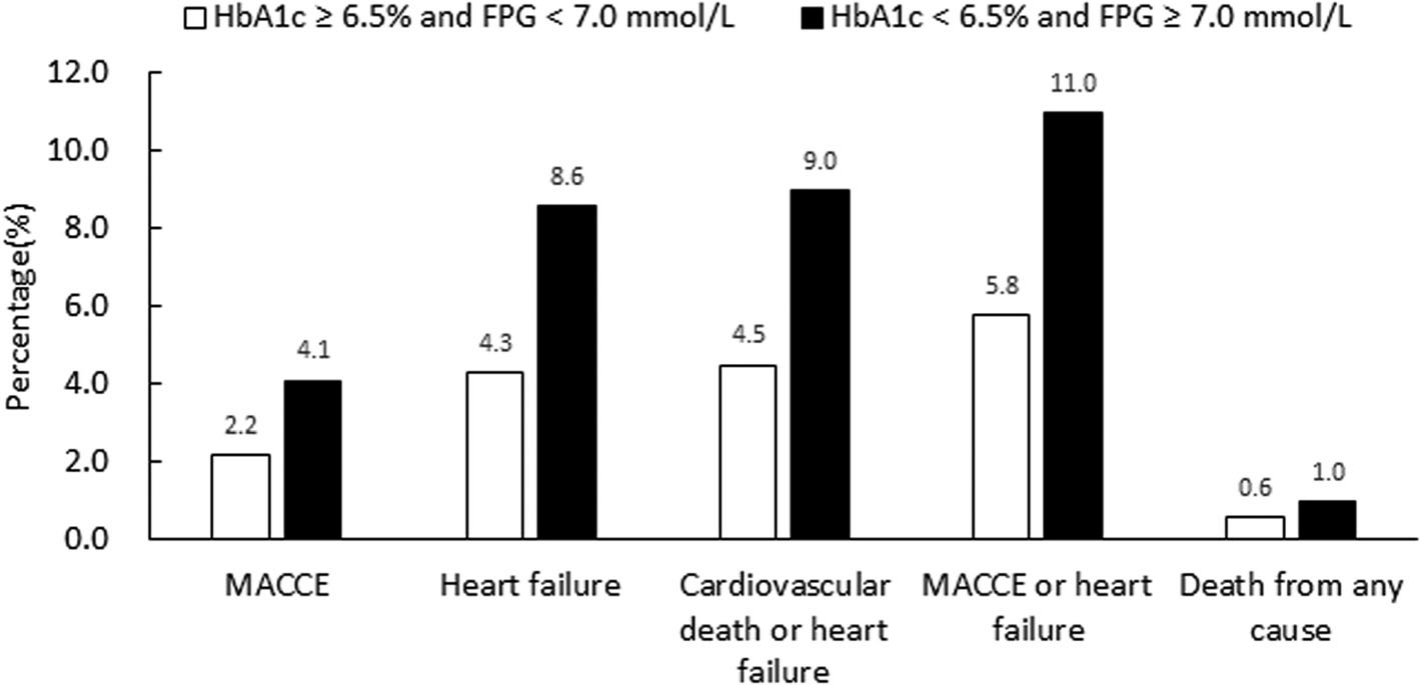
In-hospital outcomes in patients with discrepancy between HbA_1c_ and FPG. FPG, fasting plasma glucose; HbA_1c_, glycosylated hemoglobin; MACCE, major adverse cardiovascular and cerebrovascular event

The comparison of in-hospital outcomes between the increased HbA_1c_ but normal FPG group and the increased FPG but normal HbA_1c_ group is shown in Fig. 2. The rates of all the in-hospital outcomes were higher in the increased FPG but normal HbA_1c_ group than in the increased HbA_1c_ but normal FPG group. A logistic regression model was performed to explore the relationship between the type of discrepancy and in-hospital outcomes, except for death from any cause because of the small event number. In univariate logistic regression analysis, a significantly higher risk of all the in-hospital outcomes was observed in patients with increased FPG but normal HbA_1c_ (Table 2). After adjusting for confounders in the multivariate logistic regression model, patients in the increased FPG but normal HbA_1c_ group had a significant 1.6-fold increased risk of heart failure (OR, 1.62; 95% CI, 1.08–2.44), a 1.6-fold increased risk of composite cardiovascular death and heart failure (OR, 1.63; 95% CI, 1.09–2.43), and a 1.6-fold increased risk of composite MACCEs and heart failure (OR, 1.56; 95% CI, 1.08–2.24) compared to patients in the increased HbA_1c_ group (Table 2). The effect on MACCEs was not significant (OR, 1.49; 95% CI, 0.85–2.63) (Table 2). Furthermore, to investigate the association between the severe discrepancy and in-hospital outcomes, we used the cut-off values of HbA1c 7.5% and FPG 8.0 mmol/L. Although the significant *P* values were not shown in logistic regression analysis, trends that patients in the increased FPG group had higher risks of MACCEs, heart failure, composite cardiovascular dearth and heart failure, and composite MACCEs and heart failure were observed (Additional file 1: Table S3).

**Table 2:** Logistic regression analysis for in-hospital outcomes in the increased FPG group compared with the increased HbA_1c_ group^a^

	Unadjusted OR (95% CI)	*P* value	Adjusted OR (95% CI)^b^	*P* value
MACCE	1.94(1.13–3.34)	0.016	1.52(0.85–2.72)	0.158
Heart failure	2.09(1.42–3.07)	< 0.001	1.63(1.07–2.48)	0.024
Cardiovascular death or heart failure	2.11(1.44–3.07)	< 0.001	1.63(1.08–2.47)	0.021
MACCE or heart failure	1.98(1.41–2.79)	< 0.001	1.57(1.07–2.28)	0.020

^a^A categorized variable to compare the increased FPG group with the increased HbA1c group was used in logistic regression analysis

^b^ORs were adjusted for age, gender, systolic blood pressure, heart rate, current smoker, hypertension, hemoglobin at admission, eGFR at admission, Killip class, type of acute coronary syndrome, glucose-lowering drug use, and β-blocker use during hospitalization

*FPG* Fasting plasma glucose, *HbA*_*1c*_ Glycosylated hemoglobin, *MACCE* Major adverse cardiovascular and cerebrovascular event

**Fig. 3:**
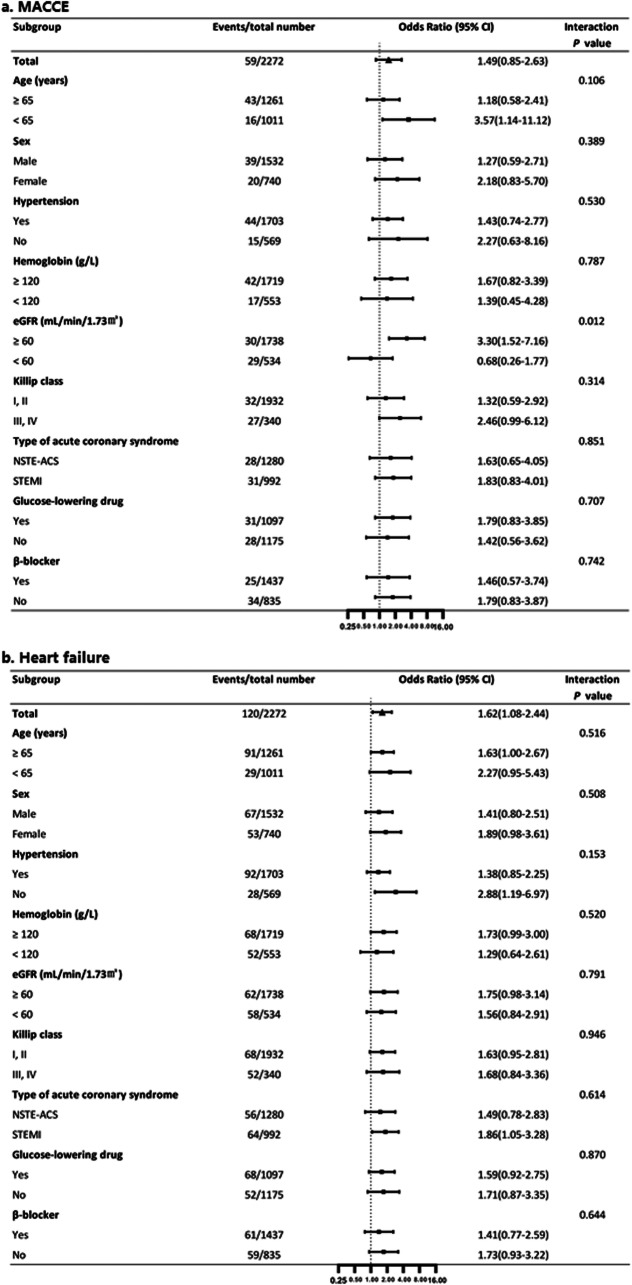
Subgroup analysis for association between the type of discrepancy and in-hospital outcomes*. * A categorized variable to compare the increased FPG group with the increased HbA_1c_ group was used in logistic regression analysis. ORs were adjusted for age, gender, systolic blood pressure, heart rate, current smoker, hypertension, hemoglobin at admission, eGFR at admission, Killip class, type of acute coronary syndrome, and glucose-lowering drug use. Panel **a** shows the effect of the increased FPG group on MACCE compared with increased HbA_1c_ group. Panel **b** shows the effect of the increased FPG group on heart failure compared with increased HbA_1c_ group. eGFR, estimated glomerular filtration rate; FPG, fasting plasma glucose; HbA_1c_, glycosylated hemoglobin; MACCE, major adverse cardiovascular and cerebrovascular event; NSTE-ACS, non-ST-segment elevation acute coronary syndrome; STEMI, ST-segment elevation myocardial infarction

**Fig. 4:**
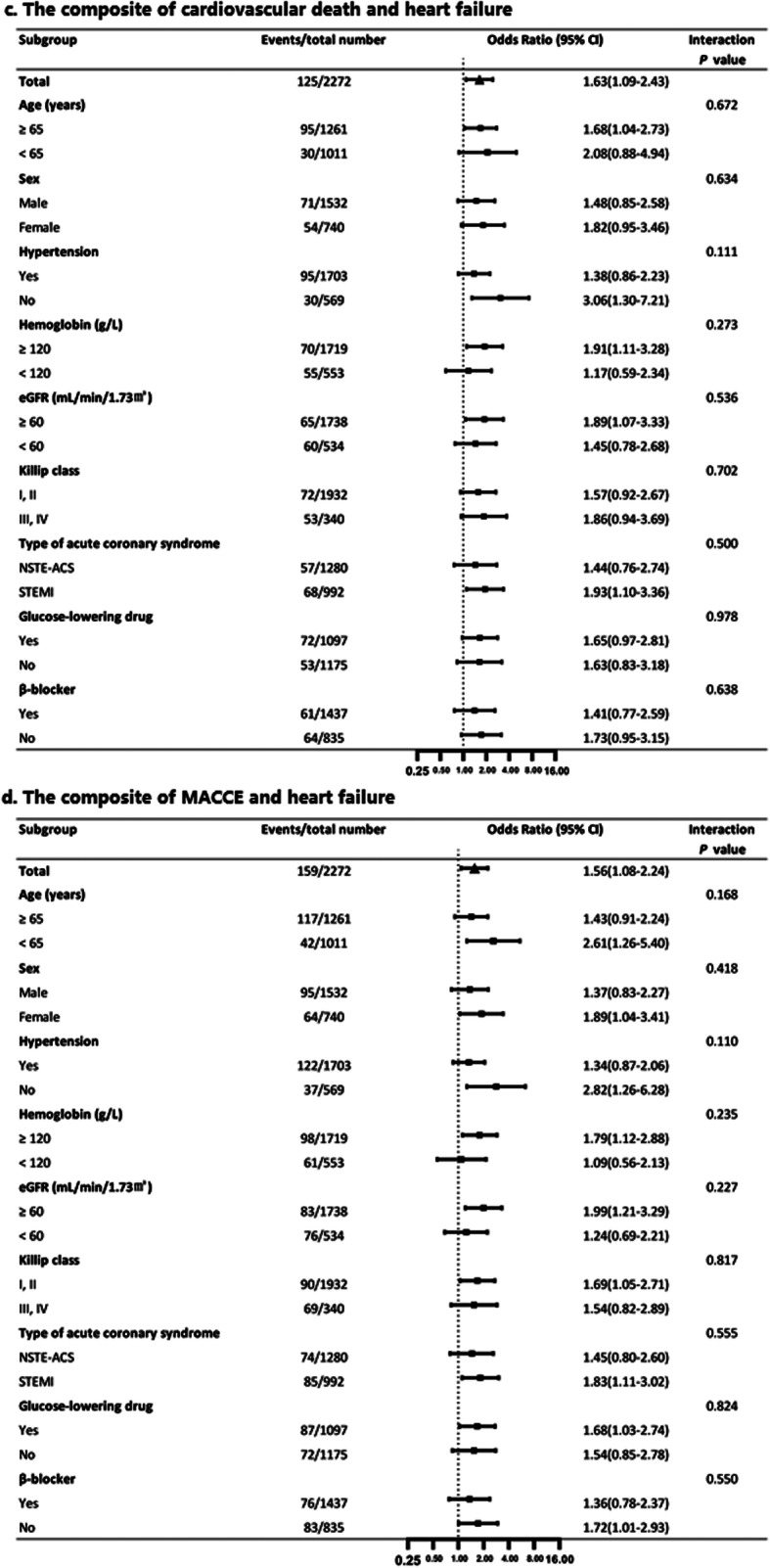
Panel c shows the effect of the increased FPG group on the composite of cardiovascular death and heart failure compared with the increased HbA_1c_ group. Panel d shows the effect of the increased FPG group on the composite of MACCE and heart failure compared with the increased HbA_1c_ group. eGFR, estimated glomerular filtration rate; FPG, fasting plasma glucose; HbA_1c_, glycosylated hemoglobin; MACCE, major adverse cardiovascular and cerebrovascular event; NSTE-ACS, non-ST-segment elevation acute coronary syndrome; STEMI, ST-segment elevation myocardial infarction

Subgroup analysis was performed based on age, sex, medical history, Killip class, hemoglobin, eGFR, type of ACS, glucose-lowering drug use before hospitalization, and β-blocker use during hospitalization. A higher risk of all the in-hospital outcomes was observed in patients with increased FPG but normal HbA_1c_, which was consistent in all subgroups (all P for interaction > 0.05), except for the eGFR subgroup for MACCEs (Figs. 3 and 4), which showed that increased FPG but normal HbA_1c_ increased the risk of MACCEs to a greater extent in patients with eGFR ≥60 ml min^− 1^ (1.73 m)^− 2^.

## Discussion

In this study, we investigated the type of discrepancy between HbA_1c_ and FPG in patients with ACS and diabetes. We found that nearly one-third of patients had a discrepancy between HbA_1c_ and FPG. Of the patients with discrepancies, the patients with increased FPG had a higher risk of in-hospital adverse cardiovascular outcomes than those with increased HbA_1c_.

Discrepancies between HbA_1c_ and FPG have been reported by some studies. A study of the risk of hypertension in patients with prediabetes demonstrated the discrepancy between HbA_1c_ and FPG [20]. A study using data from residents of Yunnan Province, China, showed that a discrepancy between HbA_1c_ and FPG was present in approximately 30% of participants [21]. In our study, the discrepancy between HbA_1c_ and FPG was also found in patients with ACS and diabetes. We found that the discrepancy group, composed of 77.5% patients with increased HbA_1c_ but normal FPG and 22.5% patients with increased FPG but normal HbA_1c_, accounted for 29% of the total study population. Patients often experience hyperglycemia in the acute phase of many diseases, such as ACS, which is called stress hyperglycemia. HbA_1c_ reflects average glycemia over approximately 3 months, so an increase in HbA_1c_ usually indicates chronic hyperglycemia. We found that patients in the increased FPG but normal HbA_1c_ group were more likely to have lower eGFR and to be treated with glucose-lowering agents. A higher proportion of glucose-lowering agent use may be related to well-controlled blood glucose and lower HbA_1c_. Furthermore, changes in the metabolism of glucose-lowering drugs, insulin clearance, and the uremic environment in patients with renal function insufficiency may also reduce HbA_1c_ values [22]. From our study, not only was a discrepancy between HbA_1c_ and FPG be found in patients with chronic kidney disease, but the proportion of the increased FPG group was found to be significantly higher than that of the increased HbA_1c_ group.

There is a strong association between cardiovascular disease, diabetes and chronic kidney disease. People with diabetes and chronic kidney disease have a substantially increased risk of all-cause mortality, cardiovascular mortality, and kidney failure [23, 24]. Furthermore, we analyzed the relationship between the type of discrepancy and in-hospital outcomes. We know that HbA_1c_ and FPG are both closely related to in-hospital outcomes. Most previous studies have shown that increased HbA_1c_ or FPG was significantly associated with poor in-hospital outcomes in patients with ACS and diabetes. An observational study that included 250 patients with ACS found that coronary atherosclerosis was more advanced in patients with HbA_1c_ ≥ 5.7% than in those with HbA_1c_ < 5.7% [17]. Goyal et al. [25] conducted a post hoc analysis including two randomized controlled trials of acute myocardial infarction with ST-segment elevation, involving 30,536 subjects with diabetes history, and showed that patients with in-hospital glucose ≥144 mg/dL had a very high risk of death. A retrospective cohort study of 768 patients with post-myocardial infarction was conducted, and the results showed that presence of impaired glucose tolerance and newly diagnosed diabetes mellitus is associated with increased incidence of adverse outcomes [26]. Kiviniemi et al. conducted a prospective cohort study that included patients with coronary artery disease, and the results showed that the adverse outcomes in patients with impaired glucose tolerance or impaired fasting glucose does not differ from those values in patients with normal glycemic status, while patients with type 2 diabetes had a higher risk of adverse outcomes [27]. Contradictory results between this and previous studies may not be fully explained, but the differences in research population and medical treatment may play a role. However, in clinical practice, some conditions, such as acute stress, renal dysfunction, and anemia, can cause uncertainty in the measured values of FPG and HbA_1c_ and the discrepancy between them. Until now, the association of in-hospital outcomes with the discrepancy between HbA_1c_ and FPG in patients with ACS and diabetes has not been clear. There are few studies focusing on this issue. From our study, we can conclude that patients in the increased FPG group, who were more likely to have a higher heart rate, poorer heart function, and higher incidence rates of STEMI and hypertension, had a higher risk of in-hospital cardiovascular adverse outcomes than those with increased HbA_1c_. Stress hyperglycemia, which is a reflection of high free fatty acids, insulin resistance, and steroid hormones, affects the course of the disease in an adverse way [28]. From another study, we learned that the level of stress hyperglycemia often correlates with the severity of disease and can predict mortality [29]. In our study, we also found that patients with severe clinical conditions, such as a higher heart rate and poorer heart function, were more likely to have increased FPG. As a result, stress hyperglycemia may have a greater adverse effect on patients with ACS and diabetes than chronic hyperglycemia.

The findings of this study may have some important implications for clinical practice. The HbA_1c_ test is a major tool for assessing glycemic control and has strong predictive value for diabetes complications [30]. Chronic hyperglycemia is an important risk factor for cardiovascular disease and mortality [24], although the variability in HbA_1c_ in patients with renal insufficiency should be considered. However, in patients with ACS and diabetes, increased FPG may be associated with a higher risk of adverse in-hospital outcomes, even if HbA_1c_ is well controlled. These patients, especially those with renal insufficiency, should be given more attention and closer monitoring in clinical practice.

The major strength of our study is that it is based on a nationally representative registry and is aimed at investigating the discrepancy between HbA_1c_ and FPG and the influence of the discrepancies on the in-hospital outcomes of patients with ACS and diabetes, which has rarely been reported until now. Our study also has certain limitations. First, all-cause mortality was not included in the logistic regression analysis because of very limited events. Second, we could not collect all information related to glucose metabolism in this real-world study of ACS patients based on medical records, thus contributing to some residual confounding from unmeasured confounders. Last, fasting status, blood sample collection and testing methods were difficult to unify, as this was a real-world multicenter study.

## Conclusions

In summary, our study showed that the discrepancy between HbA_1c_ and FPG accounts for nearly 30% of discrepancies among patients with ACS and diabetes. Patients with an increased level of FPG had a higher risk of in-hospital cardiovascular adverse outcomes than those with an increased level of HbA_1c_. This result may indicate that when HbA_1c_ and FPG are inconsistent in patients with ACS and diabetes, the increased FPG that may be caused by stress hyperglycemia may have a more substantial adverse effect than increased HbA_1c_, which may be caused by chronic hyperglycemia. These high-risk patients should be given more attention and closer monitoring in clinical practice.

## Supplementary information

**Additional file 1 **: **Table S1.** Variables with missing values and missing rates for total population in the CCC - ACS project (*N* = 92,509). **Table S2.** Prevalence of discrepancies in different populations. **Table S3.** Logistic regression analysis for in-hospital outcomes in the increased FPG group compared with the increased HbA1c group. **Table S4.** Investigators of the CCC-ACS project. **Figure S1.** Flow chart of patients considered for inclusion. **Figure S2.** Association between discrepancy and renal function.

## Data Availability

The datasets used and analyzed during the current study are available from the principal investigator of CCC-ACS on reasonable request.

## Abbreviations

ACS: Acute coronary syndrome
CCC-ACS: Improving Care for Cardiovascular Disease in China - acute coronary syndrome
eGFR: Estimated glomerular filtration rate
FPG: Fasting plasma glucose
HbA_1c_: Glycosylated hemoglobin
MACCEs: Major adverse cardiovascular and cerebrovascular events
STEMI: ST-segment elevation myocardial infarction
