# Correction to: Admission fasting plasma glucose is associated with in-hospital outcomes in patients with acute coronary syndrome and diabetes: findings from the improving Care for Cardiovascular Disease in China - Acute Coronary Syndrome (CCC-ACS) project

**DOI:** 10.1186/s12872-021-02118-y

**Published:** 2021-07-22

**Authors:** Nan Ye, Lijiao Yang, Guoqin Wang, Weijing Bian, Fengbo Xu, Changsheng Ma, Dong Zhao, Jing Liu, Yongchen Hao, Jun Liu, Na Yang, Hong Cheng, the CCC-ACS

**Affiliations:** 1 Renal Division, Beijing Anzhen Hospital, Capital Medical University, Beijing, China; 2 Department of Cardiology, Beijing Anzhen Hospital, Capital Medical University, Beijing, China; 3 Department of Epidemiology, Beijing Anzhen Hospital, Capital Medical University, Beijing Institute of Heart, Lung and Blood Vessel Diseases, No. 2 Anzhen Street, Chao yang District, Beijing, 100029 PR China

## Correction to: BMC Cardiovascular Disorders (2020) 20:380 10.1186/s12872-020-01662-3

Following publication of the original article [1], an error was identified in the text:

The original text currently reads:

A total of 104,516 inpatients with ACS, identified based on their principal diagnosis at discharge, were enrolled from 240 hospitals across China from November 2014 to December 2019.

The sentence should read:

A total of 104,516 inpatients with ACS, identified based on their principal diagnosis at discharge, were enrolled from 240 hospitals across China from November 2014 to July 2019.

The original article has been corrected as well.
