# Supplementary material for: Admission fasting plasma glucose is associated with in-hospital outcomes in patients with acute coronary syndrome and diabetes: findings from the improving Care for Cardiovascular Disease in China - Acute Coronary Syndrome (CCC-ACS) project
Source: BMC Cardiovasc Disord. 2020 Aug 20;20:380. doi: 10.1186/s12872-020-01662-3 (PMC7441713; doi:10.1186/s12872-020-01662-3)
Supplement: Supplementary file 1 — Additional file 1 : Table S1. Variables with missing values and missing rates for total population in the CCC - ACS project (N = 92,509). Table S2. Prevalence of discrepancies in different populations. Table S3. Logistic regression analysis for in-hospital outcomes in the increased FPG group compared with the increased HbA1c group. Table S4. Investigators of the CCC-ACS project. Figure S1. Flow chart of patients considered for inclusion. Figure S2. Association between discrepancy and renal function. [file 12872_2020_1662_MOESM1_ESM.pdf]

Table S1. Variables with missing value and missing rates for total population in CCC - ACS project (N=92509).

Variables	Missing rates, N (%)
Age	361(0.39)
Systolic blood pressure	161(0.17)
Diastolic blood pressure	196(0.21)
Heart rate	221(0.24)
Serum creatinine	3671(3.97)
Hemoglobin	3299(3.57)
Fasting plasma glucose	12157(13.14)
Total cholesterol	9695(10.48)
HDL-cholesterol	9515(10.29)
LDL-cholesterol	9109(9.85)
Triglyceride	9295(10.05)

CCC - ACS, The Improving Care for Cardiovascular Disease in China-Acute Coronary Syndrome; HDL, high density lipoprotein; LDL, low density lipoprotein.

Table S2. Prevalence of discrepancy in different population.

Subgroup	Increased HbA_{1c} group	Increased FPG group	*P* value
**Age (years)**			0.583
≥ 65 (n=1261)	977(78.0)	284(22.0)	
< 65 (n=1011)	784(77.1)	227(22.9)	
**Sex**			0.713
Male (n=1532)	1184(77.3)	348(22.7)	
Female (n=740)	577(78.0)	163(22.0)	
**Hemoglobin (g/l)**			0.965
≥ 120 (n=1719)	1332(77.5)	387(22.5)	
< 120 (n=553)	429(77.6)	124(22.4)	
**eGFR (ml min^{-1} $[1.73m]^{-2}$)**			0.078
≥ 60 (n=1738)	1362(78.4)	376(21.6)	
< 60 (n=534)	399(74.7)	135(25.3)	
**Killip class**			0.289
I, II (n=1932)	1505(77.9)	427(22.1)	
III, IV (n=340)	256(75.3)	84(24.7)	
**Type of acute coronary syndrome**			<0.001
NSTE-ACS (n=1280)	1045(81.6)	235(18.4)	
STEMI (n=992)	716(72.2)	276(27.8)	
**Glucose-lowering drug**			<0.001
Yes (n=1097)	809(73.7)	288(26.3)	
No (n=1175)	952(81.0)	223(19.0)	

eGFR, estimated glomerular filtration rate; FPG, fasting plasma glucose; HbA1c, glycosylated hemoglobin; NSTE-ACS, non-ST-segment elevation acute coronary syndrome; STEMI, ST-segment elevation myocardial infarction.

Table S3. Logistic regression analysis for in-hospital outcomes in increased FPG group compared with increased HbA1c group†.

	Unadjusted OR (95% CI)	P value	Adjusted OR (95% CI)*	P value
MACCE	1.94(1.02-3.69)	0.043	1.41(0.71-2.79)	0.330
Heart failure	1.46(0.90-2.38)	0.128	1.14(0.67-1.93)	0.632
Cardiovascular death or heart failure	1.49(0.93-2.40)	0.099	1.13(0.67-1.89)	0.648
MACCE or heart failure	1.59(1.04-2.44)	0.032	1.23(0.78-1.94)	0.381

† A categorized variable to compare increased FPG group (HbA1c < 6.5% and FPG ≥ 8.0 mmol/L) with increased HbA1c group (HbA1c ≥ 7.5% and FPG < 7.0 mmol/L) was used in logistic regression analysis.

* ORs were adjusted for age, gender, systolic blood pressure, heart rate, current smoker, previous acute myocardial infarction, previous coronary artery bypass grafting, hemoglobin at admission, type of acute coronary syndrome, triglyceride, high density lipoprotein-cholesterol, and glucose-lowering drug use, β-blocker use during hospitalization.

FPG, fasting plasma glucose; HbA1c, glycosylated hemoglobin; MACCE, major adverse cardiovascular and cerebrovascular event.

Table S4. Investigators of CCC-ACS project.

ID	Hospitals	Territories	Provinces	City	Investigator
1	Shanxi Cardiovascular Hospital	Northern China	Shanxi	Taiyuan	Bao Li
2	Nanjing Drum Tower Hospital, The Affiliated Hospital of Nanjing University Medical School	Eastern China	Jiangsu	Nanjing	Biao Xu, Guangshu Han
3	Hainan General Hospital	Southern China	Hainan	Haikou	Bin Li
4	The Second Hospital of Jilin University	Northeast China	Jilin	Changchun	Bin Liu
5	The 2nd Affiliated Hospital of Harbin Medical University	Northeast China	Heilongjiang	Harbin	Bo Yu
6	The Ninth Hospital Affiliated to Shanghai Jiaotong University School of Medicine	Eastern China	Shanghai	Shanghai	Changqian Wang
7	Henan Provincial People’s Hospital	Central China	Henan	Zhengzhou	Chuanyu Gao
8	Shanxi Provincial People’s Hospital	Northern China	Shanxi	Taiyuan	Chunlin Lai
9	Xinqiao Hospital, Third Military Medical University	Southwest China	Chongqing	Chongqing	Cui Bin, Lan Huang
10	China Meitan General Hospital	Northern China	Beijing	Beijing	Di Wu

11	The 309th Hospital of Chinese People’s Liberation Army	Northern China	Beijing	Beijing	Fakuan Tang, Jun Xiao
12	Zhongda Hospital, Southeast University	Eastern China	Jiangsu	Nanjing	Genshan Ma
13	The First Affiliated Hospital of Liaoning Medical University	Northeast China	Liaoning	Jinzhou	Guizhou Tao
14	Xinjiang Uygur Autonomous Region People’s Hospital	Northwest China	Xinjiang	Urumchi	Guoqing Li
15	Sir Run Run Shaw Hospital, College of Medicine, Zhejiang University	Eastern China	Zhejiang	Hangzhou	Guosheng Fu
16	Beijing Friendship Hospital, Capital Medical University	Northern China	Beijing	Beijing	Hongwei Li
17	The First Affiliated Hospital of Bengbu Medical College	Eastern China	Anhui	Bengbu	Honhju Wang
18	General Hospital of TISCO	Northern China	Shanxi	Taiyuan	Huifeng Wang
19	Dongguan People’s Hospital	Southern China	Guangdong	Dongguan	Jianfeng Ye
20	Panyu Hospital of Chinese Medicine	Southern China	Guangdong	Guangzhou	Jianhao Li
21	Peking University First Hospital	Northern China	Beijing	Beijing	Jie Jiang
22	Sun Yat-sen Memorial Hospital, Sun Yat-sen University	Southern China	Guangdong	Guangzhou	Jingfeng Wang
23	Guangdong General Hospital	Southern China	Guangdong	Guangzhou	Jiyan Chen
24	Hospital of Xinjiang Production & Construction Corps	Northwest China	Xinjiang	Urumchi	Junming Liu

25	The Military General Hospital of Beijing PLA	Northern China	Beijing	Beijing	Junxia Li
26	The First Affiliated Hospital of Guangxi Medical University	Southern China	Guangxi	Nanning	Lang Li
27	Tongren Hospital Affiliated to Shanghai Jiaotong University School of Medicine	Eastern China	Shanghai	Shanghai	Li Jiang
28	Binzou City Center Hospital	Eastern China	Shandong	Binzhou	Lijun Meng
29	The First Affiliated Hospital of Zhengzhou University	Central China	Henan	Zhengzhou	Ling Li
30	Xijing Hospital	Northwest China	Shaanxi	Xi’an	Ling Tao
31	The Affiliated Hospital of Guizhou Medical University	Southwest China	Guizhou	Guiyang	Lirong Wu
32	First Affiliated Hospital of the People’s Liberation Army General Hospital	Northern China	Beijing	Beijing	Miao Tian
33	The Second People’s Hospital of Yunnan Province	Southwest China	Yunnan	Kunming	Minghua Han
34	Haikou People’s Hospital	Southern China	Hainan	Haikou	Moshui Chen
35	Gansu Provincial Hospital	Northwest China	Gansu	Lanzhou	Ping Xie
36	The First Affiliated Hospital of Henan University of Science and Technology	Central China	Henan	Luoyang	Pingshuan Dong

37	Chenzhou First People’s Hospital	Central China	Hunan	Chenzhou	Qiaoqing Zhong
38	People’s Hospital of Qinghai Province	Northwest China	Qinghai	Xining	Rong Chang
39	Affiliated Hospital of Ningxia Medical University	Northwest China	Ningxia	Yinchuan	Shaobin Jia
40	Beijing Anzhen Hospital, Capital Medical University	Northern China	Beijing	Beijing	Shaoping Nie, Xiaohui Liu
41	North Jiangsu People’s Hospital	Eastern China	Jiangsu	Yangzhou	Shenghu He
42	Shanghai Sixth People’s Hospital	Eastern China	Shanghai	Shanghai	Shixin Ma
43	The First Hospital of Handan	Northern China	Hebei	Handan	Shuanli Xin
44	Huai’an First People’s Hospital	Eastern China	Jiangsu	Huai’an	Shuren Ma
45	The First Affiliated Hospital of Chongqing Medical University	Southwest China	Chongqing	Chongqing	Suxin Luo
46	Navy General Hospital	Northern China	Beijing	Beijing	Tianchang Li
47	Zhejiang Provincial Hospital of TCM	Eastern China	Zhejiang	Hangzhou	Wei Mao
48	The Third Xiangya Hospital of Central South University	Central China	Hunan	Changsha	Weihong Jiang
49	Affiliated Hospital of Qinghai University	Northwest China	Qinghai	Xining	Weijun Liu

50	Teda International Cardiovascular Hospital	Northern China	Tianjin	Tianjin	Wenhua Lin
51	The Second Hospital of Hebei Medical University	Northern China	Hebei	Shijiazhuang	Xianghua Fu
52	Changhai Hospital of Shanghai	Eastern China	Shanghai	Shanghai	Xianxian Zhao
53	The Second Affiliated Hospital to Nanchang University	Eastern China	Jiangxi	Nanchang	Xiaoshu Cheng
54	Hebei General Hospital	Northern China	Hebei	Shijiazhuang	Xiaoyong Qi
55	Inner Mongolia People’s Hospital	Northern China	Inner Mongolia	Hohhot	Xingsheng Zhao
56	The General Hospital of Shenyang Military Region	Northeast China	Liaoning	Shenyang	Yaling Han
57	The First Hospital of Jilin University	Northeast China	Jilin	Changchun	Yang Zheng
58	Tianjin Chest Hospital	Northern China	Tianjin	Tianjin	Yin Liu
59	Hunan Provincial People’s Hospital	Central China	Hunan	Changsha	Ying Guo
60	People’s Hospital of Yuxi City	Southwest China	Yunnan	Yuxi	Yinglu Hao
61	The People’s Hospital of Guangxi Zhuang Autonomous Region	Southern China	Guangxi	Nanning	Yingzhong Lin
62	The First Teaching Hospital of Xinjiang Medical University	Northwest China	Xinjiang	Urumchi	Yitong Ma
63	Baogang Hospital	Northern China	Inner Mongolia	Baotou	Yongdong Li

64	Tianjin Medical University General Hospital	Northern China	Tianjin	Tianjin	Yuemin Sun
65	The Second Affiliated Hospital of Zhengzhou University	Central China	Henan	Zhengzhou	Yulan Zhao
66	Nanfang Hospital of Southern Medical University	Southern China	Guangdong	Guangzhou	Yuqing Hou
67	The First Affiliated Hospital to Nanchang University	Eastern China	Jiangxi	Nanchang	Zeqi Zheng
68	The First Affiliated Hospital of Lanzhou University	Northwest China	Gansu	Lanzhou	Zheng Zhang
69	The Third Hospital of Shijiazhuang	Northern China	Hebei	Shijiazhuang	Zhenguo Ji
70	Wuxi People's Hospital	Eastern China	Jiangsu	Wuxi	Zhenyu Yang
71	Jiangsu Province Hospital	Eastern China	Jiangsu	Nanjing	Zhijian Yang
72	The Second Hospital of Shanxi Medical University	Northern China	Shanxi	Taiyuan	Zhiming Yang
73	The Affiliated Hospital of Xuzhou Medical College	Eastern China	Jiangsu	Xuzhou	Zhirong Wang
74	Southwest Hospital, Third Military Medical University	Southwest China	Chongqing	Chongqing	Zhiyuan Song
75	The First Affiliated Hospital of Xi'an Jiaotong University	Northwest China	Shaanxi	Xi'an	Zuyi Yuan
76	Yangzhou First People's Hospital	Eastern China	Jiangsu	Yangzhou	Aihua Li
77	Hospital 463 of Chinese People's Liberation Army	Northeast China	Liaoning	Shenyang	Bosong Yang

78	The Central Hospital of Mianyang	Northwest China	Sichuan	Mianyang	Caidong Luo
79	Liaocheng People's Hospital	Eastern China	Shandong	Liaocheng	Chunyan Zhang
80	Yancheng Third People's Hospital	Eastern China	Jiangsu	Yancheng	Chunyang Wu
81	The Second Xiangya Hospital of Central South University	Central China	Hunan	Changsha	Daoquan Peng
82	The Central Hospital of Panzhihua	Northwest China	Sichuan	Panzhihua	Dawen Xu
83	The First Hospital of Qiqihar City	Northeast China	Heilongjiang	Qiqihar	Gang Xu
84	The Third the People's Hospital of Bengbu	Eastern China	Anhui	Bengbu	Gengsheng Sang
85	The First Hospital of Jiamusi	Northeast China	Heilongjiang	Jiamusi	Guixia Zhang
86	Zhoushan People's Hospital	Eastern China	Zhejiang	Zhoushan	Guoxiong Chen
87	Dalian Municipal Central Hospital	Northeast China	Liaoning	Dalian	Hailong Lin
88	Renmin Hospital of Wuhan University	Central China	Hubei	Wuhan	Hong Jiang
89	Ningxia People's Hospital	Northwest China	Ningxia	Yinchuan	Hong Luan
90	The First People's Hospital of Yunnan Province (Kunhua Hospital)	Southwest China	Yunnan	Kunming	Hong Zhang
91	The Central Hospital of Zhoukou	Central China	Henan	Zhoukou	Hualing Liu

92	Anyang District Hospital	Central China	Henan	Anyang	Hui Liu
93	Sichuan Provincial People’s Hospital	Northwest China	Sichuan	Chengdu	Jianhong Tao
94	Mudanjiang Cardiovascular Disease Hospital	Northeast China	Heilongjiang	Mudanjiang	Jianwen Liu
95	Yichang Central Hospital	Central China	Hubei	Yichang	Jiawang Ding
96	Qilu Hospital of Shandong University	Eastern China	Shandong	Jinan	Jifu Li
97	Affiliated Hospital of Jiangsu University	Eastern China	Jiangsu	Zhenjiang	Jinchuan Yan
98	The First People’s Hospital of Nanning City	Southern China	Guangxi	Nanning	Jinru Wei
99	The First Affiliated Hospital of Fujian Medical University	Eastern China	Fujian	Fuzhou	Jinzi Su
100	Chengdu Third People’s Hospital	Northwest China	Sichuan	Chengdu	Jiong Tang
101	Yantaishan hospital	Eastern China	Shandong	Yantai	Juexin Fan
102	Qingdao Municipal Hospital	Eastern China	Shandong	Qingdao	Jun Guan
103	Zhongshan Hospital Affiliated to Fudan University	Eastern China	Shanghai	Shanghai	Junbo Ge
104	Longyan First Hospital	Eastern China	Fujian	Longyan	Kaihong Chen
105	Affiliated Hospital of Guangdong Medical College	Southern China	Guangdong	Guangzhou	Keng Wu

106	Jiangxi Provincial People's Hospital	Eastern China	Jiangxi	Nanchang	Lang Ji
107	Anhui Provincial Hospital	Eastern China	Anhui	Hefei	Likun Ma
108	Xiangtan City Central Hospital	Central China	Hunan	Xiangtan	Lilong Tang
109	The First Hospital of Haerbin City	Northeast China	Heilongjiang	Harbin	Lin Wei
110	Central Hospital Affiliated to Shenyang Medical College	Northeast China	Liaoning	Shenyang	Man Zhang, Kaiming Chen
111	The Central Hospital of Wuhan	Central China	Hubei	Wuhan	Manhua Chen
112	Hangzhou First People's Hospital	Eastern China	Zhejiang	Hangzhou	Ningfu Wang
113	The Central Hospital of Xuzhou	Eastern China	Jiangsu	Xuzhou	Peiying Zhang
114	The Second hospital of Dalian Medical University	Northeast China	Liaoning	Dalian	Peng Qu
115	The First Affiliated Hospital of Liaoning University of Traditional Chinese Medicine	Northeast China	Liaoning	Shenyang	Ping Hou
116	Beijing Tsinghua Changgung Hospital	Northern China	Beijing	Beijing	Ping Zhang
117	Guizhou Provincial People's Hospital	Southwest China	Guizhou	Guiyang	Qiang Wu

118	The First Affiliated Hospital of Xiamen University	Eastern China	Fujian	Xiamen	Qiang Xie
119	Quanzhou First Hospital	Eastern China	Fujian	Quanzhou	Rong Lin
120	Wuzhou People’s Hospital	Southern China	Guangxi	Wuzhou	Shaowu Ye
121	The Central Hospital of Jilin	Northeast China	Jilin	Changchun	Shuangbin Li
122	Xiangya Hospital Central South University	Central China	Hunan	Changsha	Tianlun Yang
123	Guangzhou Red Cross Hospital	Southern China	Guangdong	Guangzhou	Tongguo Wu
124	The First Affiliated Hospital of Guangzhou Medical College	Southern China	Guangdong	Guangzhou	Wei Wang
125	The First Affiliated Hospital of Wenzhou Medical University	Eastern China	Zhejiang	Wenzhou	Weijian Huang
126	The Second Affiliated Hospital of Soochow University	Eastern China	Jiangsu	Suzhou	Weiting Xu
127	Wuhan Asia Heart Hospital	Central China	Hubei	Wuhan	Xi Su
128	The First Affiliated Hospital of Soochow University	Eastern China	Jiangsu	Suzhou	Xiangjun Yang
129	Affiliated Hospital of Yan’an University	Northwest China	Shaanxi	Yan’an	Xiaochuan Ma
130	The First People’s Hospital of Jining	Eastern China	Shandong	Jining	Xiaofei Sun
131	The Central Hospital of Taiyuan	Northern China	Shanxi	Taiyuan	Xiaoping Chen

132	West China Hospital of Sichuan University	Northwest China	Sichuan	Chengdu	Xiaoping Chen
133	The Third Affiliated Hospital of Guangzhou Medical College	Southern China	Guangdong	Guangzhou	Ximing Chen
134	The First Affiliated Hospital of Wannan Medical College	Eastern China	Anhui	Wuhu	Xingsheng Tang
135	Tangdu Hospital of The Fourth Military Medical University	Northwest China	Shaanxi	Xi’an	Xue Li
136	Shanghai East Hospital Affiliated to Tongji University	Eastern China	Shanghai	Shanghai	Xuebo Liu
137	Xiamen Cardiovascular Disease Hospital	Eastern China	Fujian	Xiamen	Yan Wang
138	Zhongnan hospital of Wuhan University	Central China	Hubei	Wuhan	Yanggan Wang
139	Fujian Provincial Hospital	Eastern China	Fujian	Fuzhou	Yansong Guo
140	The First Affiliated hospital of Dalian Medical University	Northeast China	Liaoning	Dalian	Yanzong Yang
141	The First People’s Hospital of Changde	Central China	Hunan	Changde	Yi Huang
142	The First Affiliated Hospital of China Medical University	Northeast China	Liaoning	Shenyang	Yingxian Sun
143	The Fourth Affiliated Hospital of China Medical University	Northeast China	Liaoning	Shenyang	Yuanzhe Jin
144	Cangzhou Central Hospital	Northern China	Hebei	Cangzhou	Zesheng Xu
145	The Central Hospital of Shaoyang	Central China	Hunan	Shaoyang	Zewei Ouyang

146	The People’s Hospital of Liaoning Province	Northeast China	Liaoning	Shenyang	Zhanquan Li
147	The First Affiliated Hospital of Jiamusi University	Northeast China	Heilongjiang	Jiamusi	Zhaofa He
148	Tangshan Gongren Hospital	Northern China	Hebei	Tangshan	Zheng Ji
149	Huaibei Miners General Hospital	Eastern China	Anhui	Huaibei	Zhenqi Su
150	Linyi People’s Hospital	Eastern China	Shandong	Linyi	Zhihong Ou
151	Chongqing Hechuan District People’s Hospital	Southwest China	Chongqing	Chongqing	Xin Tang
152	Yuzhou City Central Hospital	Central China	Henan	Xuchang	Qinfeng Su
153	Jianshui County People’s Hospital	Southwest China	Yunnan	Honghe	Weiqing Fan
154	Dunhua City Hospital	Northeast China	Jilin	Dunhua	Fanju Meng
155	Shenyang City Electricity Central Hospital	Northeast China	Liaoning	Shenyang	Jing Xu
156	Shanghai Jingan District Shibei Hospital	Eastern China	Shanghai	Shanghai	Bin Wang
157	Beijing Fangshan District First Hospital	Northern China	Beijing	Beijing	Xuemei Peng
158	Hebei Daming County People’s Hospital	Northern China	Hebei	Handan	Haiping Guo
159	Jiangsu Binhai County People’s Hospital	Eastern China	Jiangsu	Yancheng	Yonglin Zhang

160	The First People’s Hospital of Longquanyi District	Southwest China	Sichuan	Chengdu	Wei Tuo
161	Guangxi Hengxian County People’s Hospital	Southern China	Guangxi	Nanning	Xianan Zhang
162	Hunan Changsha County First People’s Hospital	Central China	Hunan	Changsha	Siding Wang
163	People’s Hospital of Wugang	Central China	Hunan	Shaoyang	JiaoMei Yang
164	Longhui County People’s Hospital	Central China	Hunan	Shaoyang	Xiaojun Wang
165	Heilongjiang Fujin City Central Hospital	Northeast China	Heilongjiang	Jiamusi	Jiyan Yin
166	Dalian Fourth People’s Hospital	Northeast China	Liaoning	Dalian	Huifang Zhang
167	General Hospital of Guangzhou Military Command	Southern China	Guangdong	Guangzhou	Yanlie Zheng
168	The First People’s Hospital of Horqin District, Tongliao City	Northern China	Inner Mongolia	Tongliao	Junping Fang
169	Guiyang Sixth People’s Hospital	Southwest China	Guizhou	Guiyang	Kalan Luo
170	Geological Mining Hospital of Hunan Province	Central China	Hunan	Changsha	Naiyi Liang
171	Zhangzhou Municipal Hospital of Fujian Province	Eastern China	Fujian	Zhangzhou	Changyong Liu
172	Jining City Yanzhou District People’s Hospital	Eastern China	Shandong	Jining	Jian Yang
173	The People’s Hospital Feixian	Eastern China	Shandong	Linyi	Honghua Deng

174	Tangshan City Fengrun District People's Hospital	Northern China	Hebei	Tangshan	Lin Wang
175	Qian'an People's Hospital	Northern China	Hebei	Tangshan	Yuheng Yang
176	Yuzhong County People's Hospital	Northwest China	Gansu	Lanzhou	Xiaowei Peng
177	Baiyin Cite Center Hospital	Northwest China	Gansu	Baiyin	Fang Zhao
178	Mingguang People's Hospital	Eastern China	Anhui	Chuzhou	Yong Li
179	Xihua County People's Hospital	Central China	Henan	Zhoukou	Chuntong Wang
180	Zhalantun People's Hospital	Northern China	Inner Mongolia	Hulunbeier	Yuhua Zhu
181	Fengrun District Second People's Hospital	Northern China	Hebei	Tangshan	Jingshan Zhao
182	Zhangping City Hospital	Eastern China	Fujian	Zhangpin	Jinxing Yi
183	Fuqing Cite Hospital	Eastern China	Fujian	Fuqing	Ping Chen
184	The Eight Affiliated Hospital, Sun Yat-sen University	Southern China	Guangdong	Guangzhou	Nan Jia
185	The Second Affiliated Hospital of Qiqihar Medical University	Northeast China	Heilongjiang	Qiqihar	Yanli Wang
186	Wuhan University of Science and Technology Hospital	Central China	Hubei	Wuhan	Jing Hu
187	Baotou City Center Hospital	Northern China	Inner Mongolia	Baotou	Ruiping Zhao

188	Shanghai Jiading District Center Hospital	Eastern China	Shanghai	Shanghai	Xia Chen
189	Datong City Second People's Hospital	Northern China	Shanxi	Datong	Xiaoqin Zhang
190	Binyang People's Hospital	Southern China	Guangxi	Binyang	Fudong Gan
191	Deqing People's Hospital	Eastern China	Zhejiang	Deqing	Fangfang Huang
192	Xinmi people's hospital	Central China	Henan	Xinmi	Xiaolei Li
193	Dongguan Changping hospital	Southern China	Guangdong	Dongguan	Haiyun Lin
194	Gongyi people's hospital	Central China	Henan	Gongyi	Tianmin Du
195	Ye County people's hospital	Central China	Henan	Yexian	Jie Yang
196	The second people's hospital of Mengcheng	Eastern China	Anhui	Mengcheng	Pengfei Zhang
197	Nanpi People's Hospital	Northern China	Hebei	Nanpi	Hui Dong
198	Shimen People's Hospital	Central China	Hunan	Shimeng	Chuanliang Liang
199	Tieli People's Hospital	Northeast China	Heilongjiang	Tieli	Yanbo Niu
200	Sihui People's Hospital	Southern China	Guangdong	Sihui	Yuehua Huang
201	Chest Hospital of Xinjiang Uygur Autonomous Region	Northwest China	Xinjiang	Urumchi	Dongsheng Chai

202	Beian First People’s Hospital	Northeast China	Heilongjiang	Bei’an	Dongyan Li
203	Zunhua People’s Hospital	Northern China	Hebei	Zunhua	Xiaoli Yang
204	Lujiang People’s Hospital	Eastern China	Anhui	Lujiang	Qichun Wang
205	Qinyang People’s Hospital	Central China	Henan	Qinyang	Xiaowen Ma
206	Longmen People’s Hospital	Southern China	Guangdong	Longmen	Yingchao Luo
207	Quyang Renji Hospital	Northern China	Hebei	Quyang	Congliang Zhang
208	Nenjiang People’s Hospital	Northeast China	Heilongjiang	Nenjiang	Shuhua Zhang
209	Longjiang First People’s Hospital	Northeast China	Heilongjiang	Longjiang	Yuhuan Shi
210	Li County Hospital of Traditional Chinese Medicine	Central China	Hunan	Changde	Songbai Li
211	Luan County People’s Hospital	Northern China	Hebei	Luanxian	Guo Li
212	Yulong Hospital	Southwest China	Yunnan	Yulong	Zeyuan He
213	Huining People’s Hospital	Northwest China	Gansu	Huining	Jiabin Xi
214	Yuncheng Hospital	Eastern China	Shandong	Yuncheng	Jinglan Diao
215	Hepu People’s Hospital	Southern China	Guangxi	Hepu	Meisheng Lai

216	Duzishan Petrochemical Hospital	Northwest China	Xinjiang	Dushanzi	Shuqiu Qu
217	Guiding People’s Hospital	Southwest China	Guizhou	Guiding	Guoduo Chen
218	People’s Hospital of Rongchang District	Southwest China	Chongqing	Chongqing	Jie Chen
219	Ningbo First Hospital	Eastern China	Zhejiang	Ningbo	Huimin Chu
220	Ledong Second People’s Hospital	Southern China	Hainan	Ledong	Xiufeng Chen
221	Guang’an People’s Hospital	Southwest China	Sichuan	Guang’an	Tian Tuo
222	Linfen People’s Hospital	Northern China	Shanxi	Linfen	Junping Deng
223	People’s Hospital of Bozhou District	Southwest China	Guizhou	Zunyi	Shengyong Chen
224	Dianjiang People’s Hospital	Southwest China	Chongqing	Dianjiang	Yang Yu
225	First Affiliated Hospital of Harbin Medical University.	Northeast China	Heilongjiang	Harbin	Yue Li
226	Yiliang Hospital	Southwest China	Yunnan	Yiliang	Liqiong Yang
227	Haidong Ping’an District Hospital of Traditional Chinese Medicine	Northwest China	Qinghai	Haidong	Guoqin Xin
228	Ningjin People’s Hospital	Eastern China	Shandong	Ningjin	Tao Zhang
229	Yutian Hospital	Northern China	Hebei	Yutian	Xiaoyun Feng

230	Yanting People's Hospital	Southwest China	Sichuan	Yanting	Mingcheng Bai
231	The Fourth Affiliated Hospital Zhejiang University School of Medicine	Eastern China	Zhejiang	Yiwu	Shudong Xia
232	Wuxi Xishan People's Hospital	Eastern China	Jiangsu	Wuxi	Xudong Li
233	Dongfeng Hospital	Northeast China	Jilin	Dongfeng	Wei Liu
234	Zhijin People's Hospital	Southwest China	Guizhou	Zhijin	Zhongshan Wang
235	Huaiyang People's Hospital	Central China	Henan	Huaiyang	Li Wei
236	Suizhou Central Hospital	Central China	Hubei	Suizhou	Fengwei Li
237	Tonglu First People's Hospital	Eastern China	Zhejiang	Tonglu	Xiaolan Li
238	Xiantao First People's Hospital	Central China	Hubei	Xiantao	Dongmei Zhu
239	Honghu People's Hospital	Central China	Hubei	Honghu	Hong Liu
240	Xinjin County Hospital of Traditional Chinese Medicine	Northwest China	Sichuan	Xinjin	Yingbi Su

CCC-ACS: The Improving Care for Cardiovascular Disease in China-Acute Coronary Syndrome.

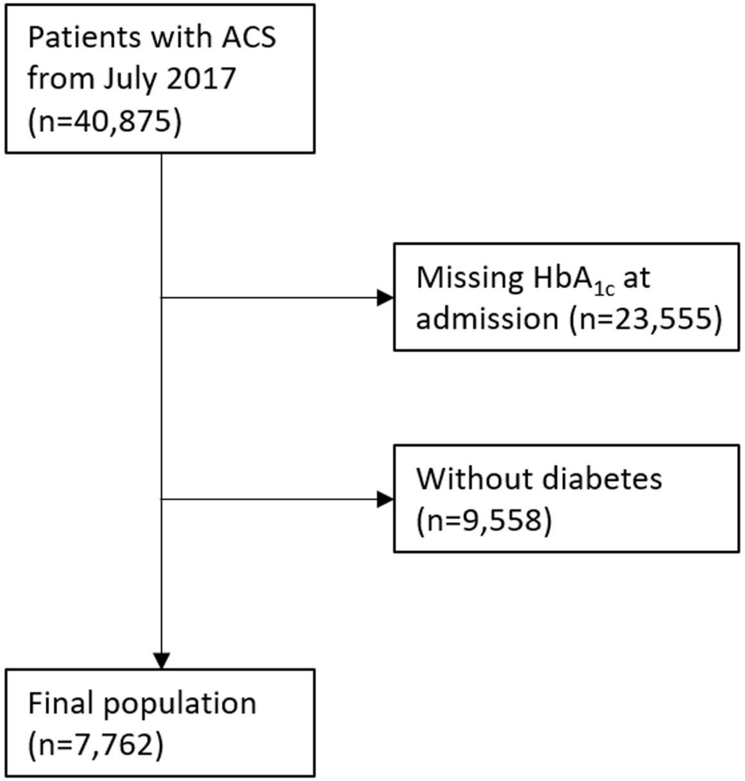

Figure S1. Flow chart of patients considered for inclusion.
ACS, acute coronary syndrome; HbA_{1c}, glycosylated hemoglobin.

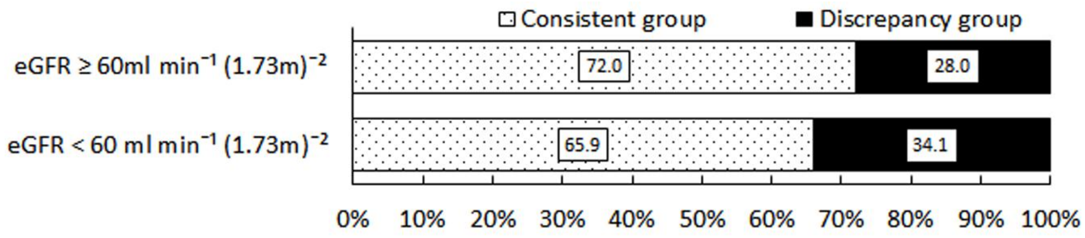

Figure S2. Association between discrepancy and renal function.
eGFR: estimated glomerular filtration rate.
